# The incidence of postoperative vasopressor usage: protocol for a prospective international observational cohort study (SQUEEZE)

**DOI:** 10.1186/s13741-023-00296-1

**Published:** 2023-03-24

**Authors:** Ben Creagh-Brown, Hannah Wunsch, Peter Martin, Pierre Harlet, Lui Forni, S. Ramani Moonesinghe, Ib Jammer

**Affiliations:** 1 Intensive Care Unit, Royal Surrey Hospital NHS Foundation Trust, Guildford, Surrey UK; 2 Department of Clinical and Experimental Medicine, Faculty of Health and Medical Sciences, University of Surrey, Guildford, UK; 3 Department of Critical Care Medicine, Sunnybrook Health Sciences Centre, Toronto, Canada; 4 Department of Applied Health Research, University College London, London, UK; 5 European Society of Anaesthesiology and Intensive Care, Brussels, Belgium; 6 Division of Surgery and Interventional Science, University College London, London, UK; 7 Department of Anaesthesia and Intensive Care, Haukeland University Hospital, Bergen, Norway; 8 Department of Clinical Medicine, University of Bergen, Bergen, Norway

**Keywords:** Vasopressor, Perioperative, Multicentre, Observational, Postoperative, Incidence

## Abstract

**Background:**

Postoperative hypotension is common after major non-cardiac surgery, due predominantly to vasodilation. Administration of infused vasopressors postoperatively may often be considered a surrogate indicator of vasodilation. The incidence of postoperative vasopressors has never been described for non-cardiac surgery, nor have outcomes associated with their use. This paper presents a protocol for a prospective international cohort study to address these gaps in knowledge.

The primary objectives are to estimate the proportion of patients who receive postoperative vasopressor infusions (PVI) and to document the variation in this proportion between hospitals and internationally. Furthermore, we will identify factors in variation of care (patient, condition, surgery, and intraoperative management) associated with receipt of PVI and investigate how PVI use is associated with patient outcomes, including organ dysfunction, length of hospital stay, and 30-day in-hospital mortality.

**Method:**

This will be a prospective, international, multicentre cohort study that includes all adult (≥ 18 years) non-cardiac surgical patients in participating centres. Patients undergoing cardiac, obstetric, or day-case surgery will be excluded. We will recruit two cohorts of patients: cohort A will include all eligible patients admitted to participating hospitals for seven consecutive days. Cohort B will include 30 sequential patients per hospital, with the single additional inclusion criterion of postoperative vasopressor usage. We expect to collect data on approximately 40,000 patients for cohort A and 12,800 patients for cohort B.

**Discussion:**

While in cardiac surgery, clinical trials have informed the choice of vasopressors used to treat postoperative vasoplegia; there remains equipoise over the best approach in non-cardiac surgery. Our study will represent the first large-scale assessment of the use of vasopressors after non-cardiac surgery. These data will inform future studies, including trials of different vasopressors and potential management options to improve outcomes and reduce resource use after surgery.

**Trial registration:**

ClinicalTrials.gov Identifier: NCT03805230, 15 January 2019.

**Supplementary Information:**

The online version contains supplementary material available at 10.1186/s13741-023-00296-1.

## Background

Postoperative hypotension is a common occurrence following major non-cardiac surgery (Briesenick et al. 2021). Clinicians routinely evaluate patients to determine the cause(s) and start appropriate therapy. Postoperative hypotension is commonly due to a combination of decreased preload (typically due to relative hypovolaemia, potentially from bleeding or fluid redistribution) or decreased afterload. Less commonly, there may be impaired cardiac contractility. Decreased afterload, otherwise known as vasodilation, is commonly due to drug effects, neuraxial anaesthesia, or systemic inflammation, and it may be resistant to treatment or prolonged (Lambden et al. 2018).

It is uncertain if vasoplegia best describes the end of the spectrum of vasodilation. It may be a pathophysiologically distinct entity representing an uncontrolled failure of vascular homeostasis. Although most common after cardiac surgery, vasoplegia also occurs after major non-cardiac surgery, mainly when there has been significant bleeding and transfusion (Lambden et al. 2018). Cardiac output is not often measured postoperatively, but when measured, postoperative vasoplegia is characterised by low systemic vascular resistance in the presence of a normal or raised cardiac output.

Once hypovolaemia has been excluded as a significant contributing factor in hypotension, typically through the administration of intravenous fluids, it is common to use vasopressor drugs (also known as vasoconstrictors) to counteract vasodilation. Intermittent dosing of short-acting drugs (“bolus” therapy) has obvious disadvantages, and therefore, many clinicians use infusions of vasopressors.

Epidural anaesthesia is well recognised to cause vasodilation, which is commonly countered through low-dose vasopressor infusions. Postoperative patients receiving higher doses of vasopressor infusions to maintain an adequate mean arterial pressure (MAP) can reasonably be described as experiencing postoperative vasoplegia. The main limitation is that excluding hypovolaemia is a prerequisite — but there is no absolute method to determine if this has been achieved. For this study, receipt of infused vasopressors is considered a surrogate indicator of significant vasodilation. In some healthcare environments, using vasopressors in the postoperative period to support blood pressure following optimisation of fluid status is commonplace. The incidence of receipt of postoperative vasopressor infusions (PVI) in non-cardiac surgery patients has never been described.

### Rationale for this study

**Fig. 1:**
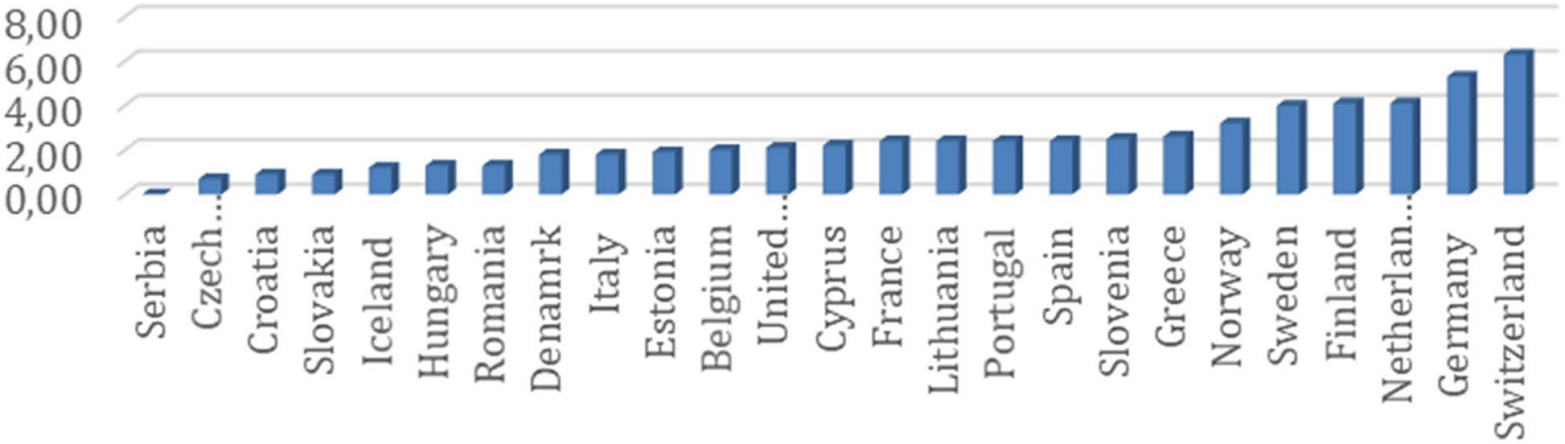
Unpublished secondary analysis of EuSOS data. Receipt of infused vasopressor or inotrope within 24 h of surgery

Preliminary data suggest that there is substantial variation in the management of postoperative hypotension between centres, countries, and continents. We obtained data from the European Surgical Outcomes Study (EuSOS) (Pearse et al. 2012), which studied 46,539 patients, including 3599 who were treated postoperatively in a critical care unit. The case report form included information about postoperative vasopressor (and inotrope) usage, which had not previously been analysed or reported. With permission, we performed a secondary analysis of these data and found that 2.7% of patients received either a vasopressor or inotrope within 24 h of surgery. There was considerable variation between countries (from 0.0 to 6.3%). A total of 75% of these patients were admitted to a critical care environment, and the most common vasoactive drug used was noradrenaline (Fig. 1).

Between July 2018 and February 2019, we invited all members of ESAIC and ESICM, respectively, to participate in a “micro-survey” that asked five concise questions. We received 2052 complete responses from 102 countries and found that 22% of respondents indicated that they encounter patients receiving postoperative vasopressor infusions “frequently” in non-cardiac surgery patients and 58% that they did so “occasionally”. A total of 20% of respondents considered PVI receipt a rare event. As well, the vasopressors used most often, in decreasing order of frequency, were noradrenaline/norepinephrine and phenylephrine.

The *EuSOS data also indicate* variation in assessment (cardiac output and invasive monitoring), environment (postoperative care units, high-dependency units, ICUs), and management (use and choice of fluids and vasopressors/inotropes) of hypotension.

In contrast to septic shock, there is no uniform definition of postoperative vasoplegia. Administration of any amount of vasopressor would provide an objective dichotomous definition, but a limitation would be the inability to differentiate degrees of vasodilation. Using a threshold dose of infused vasopressor to determine a definition is uncomfortably arbitrary.

There have been trials of different vasopressors to treat postoperative vasoplegia in cardiac surgical patients (Hajjar et al. 2017). In non-cardiac patients, PVI receipt may well be associated with patient outcomes, including incidence of organ dysfunction, organ support use, duration of hospital stay, and mortality. In order to inform future trials of methods for managing postoperative vasoplegia in non-cardiac patients, sound descriptive data on current practice and outcomes are needed. This paper presents a protocol for a study aimed at providing those data.

## Aims of the study

SQUEEZE is a prospective, international, multicentre, observational cohort study (ClinicalTrials.gov ID: NCT03805230). Our primary aims are to estimate the proportion of non-cardiac surgery patients who receive PVI and to document the variability of PVI use internationally and between hospitals (but not between practitioners).

Secondary aims are as follows: (1) to determine characteristics associated with receipt of PVI — patient, condition, surgery, and intraoperative management; (2) to estimate associations between PVI use and patient outcomes, including mortality, organ dysfunction, and length of hospital stay; and (3) in patients receiving PVI, determine the dose, therapy duration and clinical outcomes.

We want to answer the following research questions:

- (1) What proportion of patients receive PVI?
- (2) Is there variation in PVI use between different healthcare environments?
- (3) What factors (patient, condition, surgery, and intraoperative management) are associated with receiving postoperative vasopressor infusions?
- (4) What are the associations of PVI use with clinical outcomes, including 30-day mortality, organ dysfunction, and length of hospital stay?
- (5) Is there variation in practice between patients, hospitals, and countries in managing patients with PVI following surgery?
- (6) Are these variations in practice associated with clinical outcomes?
- (7) What is the health-economic impact associated with postoperative vasopressor therapy?

We note that the associations between PVI use and outcomes in our observational data won’t enable us to estimate an effect of PVI on these outcomes, since there is likely to be confounding by indication. However, the sizes of the associations we will find may be informative for future studies that wish to rigorously investigate the effect of PVI on outcomes, including randomised controlled trials.

## Methods

### Definitions

It is infusion of a drug with a predominant vasoconstrictor effect (vasopressor) (Table 1). Therefore, repeated dosing of intravenous boluses is excluded, and infusion of a drug that is predominantly a positive inotrope (without concurrent vasopressor) is excluded. Additionally, we are not interested in vasopressor infusions used intraoperatively to counter the effect of general anaesthesia (or regional anaesthesia). Because this effect can take time to resolve, any vasopressor infusion in the first hour following surgery is excluded — unless it continues after 1 h following surgery. Infusions of vasopressors started more than 24 h after the end of surgery are also excluded from this definition. Infusions of vasopressors that start before surgery will only be included if they meet the above criteria.

**Table 1:** Vasoactive drugs, grouped according to predominant action. We accept that many drugs have mixed actions

Vasopressor	Not predominantly vasopressor
• Dopamine • Epinephrine (adrenaline) • Metaraminol • Norepinephrine (noradrenaline) • Phenylephrine • Vasopressin or terlipressin • Akrinor® • Angiotensin II	• Atropine • Dobutamine • Ephedrine • Etilefrine • Glycopyrronnium • Nitrates • Milrinone

### Study population

We will recruit two cohorts of patients. The logistics of delivery are described in detail in Additional file 1. For the planned timeline, see Table 2.

**Table 2:**
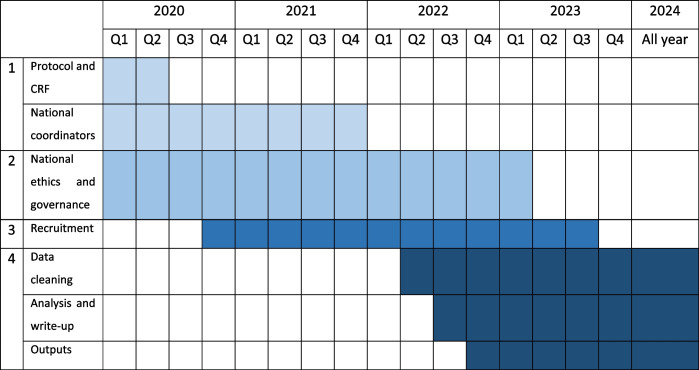
Milestones and planned timelines

*Cohort A* will include all patients admitted to participating hospitals for seven consecutive days with the following inclusion and exclusion criteria:

- Inclusion criteria: (1) Undergoing surgery (both planned and unplanned), (2) planned overnight stay at the hospital, and (3) age ≥ 18 years on the day of surgery
- Exclusion criteria: (1) Cardiac surgery; (2) obstetric surgery; (3) transplant surgery; (4) preoperatively long-term infusions of vasoactive drugs, such as epoprostenol (prostacyclin); (5) mechanical circulatory support: ventricular assist device, intra-aortic balloon pump artificial heart or similar; and (6) already been enrolled in SQUEEZE.

*Cohort B* will include 30 sequential patients from each hospital with a single additional inclusion criterion: postoperative vasopressor Infusion (PVI) — as defined above.

### Recruitment and screening

- *Cohort A*: Collecting consecutive patient data during a 7-day period will require significant human resources. Therefore, the PI will identify a suitably qualified team available on the preselected start time/date. Given the inclusive entry criteria, most patients scheduled for surgery will be eligible. We anticipate that only a minority (< 5%) of patients in cohort A will receive PVI.
- *Cohort B*: PI and the study team will actively look for patients who fulfil the criteria for cohort B (i.e. those receiving PVI). Depending upon local practice and case mix, this could take months — a maximum period of 12 months or until 30 patients are recruited, whichever occurs first. Centres that wish to recruit more than 30 patients will be permitted so.

There is no rule regarding the order in which cohorts A and B data should be collected. Centres can start recruiting to cohort B and decide when to recruit cohort A — as long as it is completed within 12 months after starting cohort B recruitment.

**Fig. 2:**
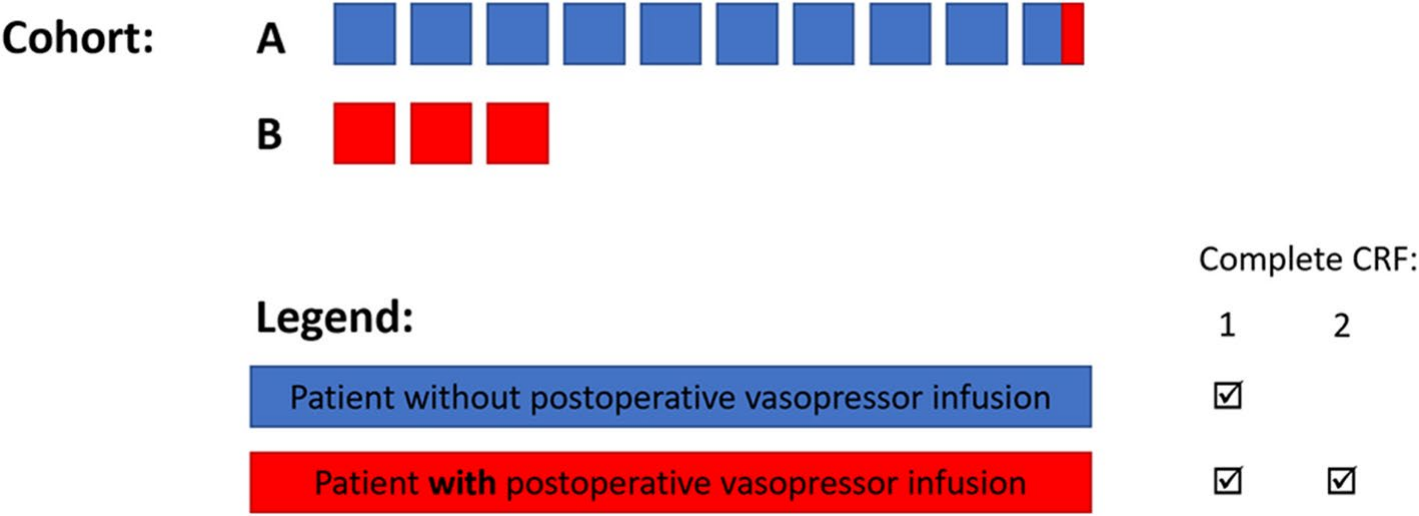
Difference between cohorts A and B and CRF1 and 2

All patients will have data collected and entered into CRF1. Patients receiving PVI will have additional information collected and entered into CRF2 (Fig. 2). Every institution that intends to recruit patients in the study will complete an “institutional survey” to allow characterisation of the healthcare environment.

Case report forms 1 and 2 are included as Additional file 2. The CRFs are transcribed into an electronic CRF (eCRF) hosted by ESAIC CTN.

### Assessments of primary endpoint outcome

#### Cohort A

For cohort A, the primary endpoint is the receipt of PVI.

#### Patients receiving PVI (cohort B and those in cohort A with PVI)

For patients receiving PVI, the primary endpoint will be death before discharge from the acute care hospital, censored at day 30.

### Assessments of secondary endpoint/outcome(s)

For all patients, secondary outcomes include organ dysfunction and length of hospital stay. These are recorded in CRF1 (Additional file 2).

### Assessment of risk factors and other patient characteristics

- Assessment of factors (patient, condition, surgery, anaesthesia) potentially predisposes to PVI — detailed in CRF1 (all from A).
- The population that receives vasopressors (some from A, all from B) is detailed in CRF2 (Additional file 2). This includes the type of vasopressor used, the dose prescribed, and the duration of vasopressor use.

No additional testing, assessments, or evaluations will be conducted. These outcomes are purely ascertained from the medical records scrutinising events during the index admission. As a pragmatic study, we will collect outcome data prioritising ease of collection and being as objective as possible. For example, we would be interested in knowing the type of postoperative pulmonary complications, according to recent standardised endpoints in perioperative medicine definitions, or the EPCO definitions; it is easier for the local investigator to ascertain “invasive mechanical ventilation / NIV / both / neither”.

### Dataset

An accurate data set will be fundamental to the success of the investigation. We have identified the critical data points while not discouraging centres from participating through an excessive burden of data collection. NC may request the addition of a limited number of data points to support the international SQUEEZE data collection and for subsequent national analyses. All additional data points must be discussed with the SSC.

Centre-specific data will be collected once for each hospital, including secondary/tertiary centres, number of operating rooms, and number and level of critical care beds.

### Assessment of optional endpoints

In some countries, the NC will liaise with the SSC, and it will be agreed that additional endpoints can be collected; a country-specific protocol will be written. For example, in the UK, we will have the additional endpoints of long-term mortality: vital status up to 5 years following surgery, from the NHS central database.

### Translational studies

Similarly, we encourage NCs to consider adding biological sample collection or physiological assessments to one or more recruiting centres in their country. The SSC will consider all requests and potentially support applications for additional funds to facilitate the delivery of such studies.

Local or national cohorts addressing additional questions and collecting additional data while sharing part of the variables collected for SQUEEZE are allowed under the following conditions: nomination of a separate sponsor (i.e. other than the ESAIC), separate ethical approval, separate informed consent, independent data management, and approval of a detailed study proposal by the SSC. The sponsor and the SSC have the right to veto the nesting of a study. For transparency, the original paper should be referenced in all articles of additional analyses. Authorship rules for potential publications derived from such additional cohort studies are to be submitted to the sponsor and SSC together with the study proposal.

### Methods of minimising bias

Selection bias will be limited through waived consent processes where applicable. The short period of data collection for Cohort A is designed to enable participating hospitals to collect data on all eligible patients within the data collection week. This method has an excellent chance of resulting in a representative sample. Information bias will be limited using a robust case report form with clearly specified definitions. A pre-specified statistical analysis plan will ensure that type 1 error inflation through multiple hypothesis tests is minimised and controlled.

## Statistical methodology and data handling

### Sample size

We aspire to collect data from at least 400 hospitals and expect the average number of patients recruited for cohort A will be about 100 per hospital. Thus, we expect to collect data for 40,000 patients in cohort A. Our secondary analysis of EuSOS (unpublished, see pilot data 1 in the introduction) showed an average use of vasoactive drugs of 2.7%. Assuming that a lesser proportion of these drugs were inotropes, we estimate 2% vasopressor usage. This estimate shows that around 800 patients will receive PVI (95% *CI*: between 745 and 855 patients, assuming a binomial distribution). We expect to have sufficient events for exploratory analyses investigating several potential risk factors of vasopressor use and their potential interactions.

For cohort B, we estimate a sample of 12,000 patients (30 patients from each of the 400 hospitals). Thus, we expect our total sample of patients with PVI to be around 12,800 (12,000 from cohort B plus 800 who were in cohort A but also received PVI and had CRF2 completed). This should result in an adequate sample to provide robust estimates of the duration of vasopressor use and allow for exploratory analyses around the timing of cessation and associated outcomes.

### Main analyses

A detailed statistical analysis plan is provided as Additional file 3. This is an exploratory study of an extensive data set based on a self-selected set of hospitals. Although our sampling procedures give us a good chance of achieving a representative sample of patients within each participating site, we do not claim to be able to achieve a random sample of hospitals from participating countries or a representative sample of patients for any country as a whole. Thus, a thorough description and graphical representation of the data will be an essential analysis method, often taking precedence over inferential procedures. As outlined below, some statistical models will be employed to aid the description and estimation of essential parameters. We will summarise patient characteristics using means, standard deviations, medians, interquartile ranges, and percentages as appropriate.

For cohort A, we will summarise the primary endpoint as a percentage of patients who receive PVI. We will also describe the variation in PVI use between hospitals and countries. Mixed-effects logistic regression will be used to document this variation employing a shrinkage estimator (best linear unbiased prediction) to control for regression to the mean and caterpillar plots.

Using patients from cohorts A and B, we will assess the relationship between PVI and potential risk factors using bivariate odds ratios. We will use multivariate mixed-effects regression with a set of plausible predictor variables to assess which are most strongly associated with receiving PVI. A shrinkage method (penalised regression, such as lasso) will be applied to the regression model to reduce the type 1 error rate and the risk of inflated estimates of the strength of associations.

Using patients from both cohorts A and B, we will assess the relationship between PVI and in-hospital mortality and secondary outcomes, using logistic regression and other statistical models as appropriate. Once again, we will use a shrinkage method to avoid overfitting. We will also describe variations in these outcomes between hospitals and countries.

Using patients from both cohorts A and B, we will graph the duration of vasopressor use using Kaplan–Meier curves and assess for any clear cut-offs to create a definition of prolonged vasopressor use. We will assess the relationship between patient characteristics (including comorbidities) and duration of vasopressor use using survival analysis. We will summarise the frequency of organ dysfunction based on different durations of postoperative vasopressor use and associated mortality. The purpose of these analyses is to document observed associations to inform future randomised trials that may wish to assess the effect of vasopressor use on outcomes.

### Identifying subcohorts

In our statistical model predicting PVI use by pre-and intraoperative variables, we will distinguish three groups of patients:

- Patients receiving epidural anaesthesia
- Patients receiving spinal anaesthesia
- All other patients

For details, consult the statistical analysis plan (see Additional file 3).

### Handling of missing data or inadequate patient recruitment

We will exclude patients from either cohort if the data is of insufficient quality or completeness. Similarly, we will exclude centres (and all the patient data from those centres) if the number of patients recruited is insufficient. Proportions of missing values will be documented for each variable individually and the data set as a whole. We will examine the need for and appropriateness of multiple imputations of other missing data based on the final data collected and based on an assessment of the likely processes that caused observation to be missing.

### Data quality

The sponsor is responsible for implementing and maintaining quality assurance and quality control systems with written SOPs to ensure that the study is conducted and data are generated, documented (recorded), and reported in compliance with the protocol, GCP, and the applicable regulatory requirements. Quality control measures will be applied to each stage of data handling to ensure that all data are reliable and have been processed correctly, including written SOP (in English for all countries) for data collection and entry, automated consistency checks, and training of NC and local PI. With support from the study coordinating office, it will be the responsibility of the NC to train local PI. Local PI will ensure that the data in the eCRF is carefully entered and verified regularly. It will be the responsibility of local PIs to conduct periodic and random checks to ensure data quality in her/his centre. The sponsor has the right to make random assessments of centres to confirm that there is no improper and incorrect data entered into the eCRF. On-site monitoring visits by the sponsor are not planned.

The sponsor is responsible for securing agreement from all involved parties to ensure direct access to all trial-related sites, source data/documents and reports for monitoring and auditing by the sponsor, and inspection by domestic and foreign regulatory authorities. Any agreements made by the sponsor with the investigator/institution and any other parties involved with the study will be in writing, as part of the protocol or in a separate agreement. No fee or financial compensation is given to PI or participating institution for patient recruitment.

### Data handling and record keeping/archiving

Data will be entered into a secure online database protected by personalised and confidential usernames and passwords, documenting the time and individuals entering the data. The language of the online database, eCRF, and the relative SOPs is English and will not be translated into the national languages. Data will be collected directly from source documents into the encoded paper CRF and secondarily entered into the eCRF. A copy of the source documents will be stored within a locked cabinet/office accessible to authorised personnel only under local and national regulations. An identifiable patient data page reporting the assigned patient identification code will be stored separately also in a locked cabinet/office (accessible to authorised personnel only) to record in-hospital outcomes, supply missing data points, and allow potential monitoring visits by national coordinating investigators, sponsor, IRB, or regulatory authorities. A signed ICF to document that written informed consent was obtained will be stored as described above. All study documents will be archived as required by local legislation.

Sponsors and centres will maintain and update their trial master files according to the ICH-GCP guidelines E6(R2) recommendation. All collected data will remain the property of the sponsor.

### Confidentiality and data protection

To safeguard patients’ confidentiality, a patient identification code will be assigned to encode data. The confidential log linking patient identification code and identifiable patient data will be stored separately in a locked cabinet accessible to authorised personnel only, and personalised and confidential usernames and passwords will protect corresponding electronic files. eCRFs are identified through the patient identification code and will not include names, initials, date of birth or local hospital patient numbers; therefore, no patient-identifiable data will be directly accessible from the eCRF. Data protection will be guaranteed through encoding and using a secured database with restricted access by individual log-in and graduated user rights. Furthermore, only encrypted data will be stored centrally. The database will be hosted on servers physically located in the European Union. Data can only be transferred to servers located in member states of the European Union or in other countries where the level of personal data protection has been determined as adequate by the European Commission based on the General Data Protection Regulation (GDPR, Article 45).

Open direct access to all relevant study information, and source data/documents, will be permitted for monitoring, audits or inspections to the sponsor, national coordinators, IRB, or regulatory authorities. All handling of personal data will comply with the GCP guidelines and strictly follow the legal and national requirements of GDPR. Please contact the ESAIC Data Protection Officer at *privacy@esaic.org* or 24 Rue des comédiens 1000 Brussels, Belgium, for any additional questions.

## Discussion, ethical, and regulatory aspects

The research project will be carried out following the research plan and the principles enunciated in the current version of the Declaration of Helsinki (Amendment 2013) by the World Medical Association and the ICH-GCP guidelines E6(R2). Specific national and local regulatory authority requirements will be followed as applicable.

### Risk categorisation

SQUEEZE is a prospective cohort study collecting clinical data on non-cardiac surgery patients. No research-related interventions are anticipated, and all patients will receive routine care according to the standards laid out in each institution. There is no risk of study participation contributing towards any adverse events. Therefore, there will be no reporting of adverse events.

Some countries may choose to nest within this study additional assessments of patient outcome or add biological sample collection or physiological assessments to add translational aspects — details of such studies will be provided outside this primary protocol.

### Institutional review board (IRB) or equivalent

In all cases, prior to study initiation, the local principal investigator (PI) at each centre must liaise with the national coordinator (NC) and ensure that they have taken the appropriate steps to seek authorisation from relevant national/regional/local bodies to permit appropriate research study conduct. No substantial changes will be made to the protocol without prior IRB approval, except where necessary to eliminate apparent immediate hazards to study participants.

### Participant information and informed consent

There are three anticipated approaches as follows:

1. This study may be considered to constitute research that requires individual patient consent.
2. In some countries, it may be possible to successfully seek a waiver of individual patient consent from an appropriate regulatory authority — in the UK; this is the Confidentiality Advisory Group (CAG) of the Health Research Authority (HRA).
3. In some countries, this may be permissible without consent; as there is no intervention, the data is being collected routinely, and only fully pseudonymised data leaves the hospital.

The SSC consider that the ideal approach is waived informed consent (2, above) because it minimises the risk of introducing selection bias. This approach was used in the UK national study SNAP-2 in 2017 and globally in the International Surgical Outcomes Study (Moonesinghe et al. 2017; International Surgical Outcomes Study G 2016). Patients at increased risk of receiving postoperative vasopressors are likely to be more severely unwell, possibly with delirium, and possibly having emergency surgery — all conditions predispose to difficulties obtaining informed consent. Therefore, by mandating individual informed consent, we might systematically exclude patients of the most significant interest and consequently undermine the generalisability of our findings. Significant differences between participants and nonparticipants may threaten the validity of results from observational studies (Kho et al. 2009).

Consent procedures and provision of patient information will be conducted following local practice. If consent is required, it will be obtained as follows: prior to surgery, the patients will be presented with the IRB-approved ICF providing sufficient time and information for the participant to make an informed decision about their participation in the study, i.e. explaining the nature of the study, its purpose, the procedures involved, the expected duration, the potential risks and benefits and any discomfort participation may entail.

In emergency surgery, when there may not be enough time to collect consent or a patient may not be able to give consent, according to the principal investigator’s judgement, consent may be obtained after surgery. In this case, consent must be obtained within 7 days of surgery or as deemed appropriate by principal investigator. Patients included in cohort B can also give their consent after surgery; this is because it will not be known if a patient is eligible for the study until during surgery, and at some sites, it may not be possible to take consent from all patients undergoing surgery (especially as the percentage of eligible patients is expected to be small).

Each participant will be informed that participation in the study is voluntary, and that he/she may withdraw from the study at any time and without explanation. Withdrawal of consent will not affect his/her subsequent medical assistance and treatment, and no further data will be collected. While already collected, encoded data will be pseudonymised, and analysis may be performed up to the point of data collection.

The participant will be informed that his/her medical records will be examined by authorised individuals other than their treating physician. The participant will read and consider the statement, have the opportunity to ask questions before signing and dating the ICF and be given a copy of the signed document. Patients will confirm that they were given adequate time to reach a decision. The ICF must also be signed and dated by the investigator (or designee) and will be retained as part of the study records.

### Participant privacy

The investigator affirms and upholds the principle of the participant’s right to privacy and shall comply with applicable privacy laws. Expressly, the anonymity of the participants shall be guaranteed when presenting the data at scientific meetings or publishing them in scientific journals.

Individual subject medical information obtained because of this study is considered confidential, and disclosure to third parties is prohibited. Subject confidentiality will be further ensured by utilising subject identification code numbers, and only pseudonymised data will be recorded in the central database.

For data verification purposes, authorised representatives of the sponsor or an ethics committee may require direct access to parts of the medical records relevant to the study, including participants’ medical history.

### International considerations

This study will permit individuals from any country to express an interest in participation. Providing they can satisfy the SSC and ESAIC, they can deliver the study following appropriate standards and sample a representative population from several hospitals within that country.

However, as this study is funded and supported by a European organisation, the priority is to consider healthcare environments that are most similar to Europe (acknowledging that within Europe, there is a degree of variation). Patient data from patients in all countries within the Council of Europe (47 member states), Canada and the USA, Australia, and New Zealand will be analysed and reported in the main manuscript.

Information from other continents (Africa, Asia, and South America) is no less valuable but will be reported separately to avoid considering incomparable healthcare environments together. For example, comparing patients enrolled in the African Surgical Outcomes Study (ASOS) (Biccard et al. 2018) with those in the Europe Surgical Outcomes Study (EuSOS) (Pearse et al. 2012) demonstrates significant differences.

Country-level datasets will be compared and presented sensitively and with suitable emphasis on the inherent limitations of these comparisons, including international differences in patterns of surgical disease and genetic backgrounds, as well as in healthcare systems. Comparisons will be made between countries grouped by income status (high/middle/low income, according to worldbank.org), but we also accept significant limitations to this methodology.

Suppose there is sufficient interest from a continent with an identified suitably experienced leader who wishes to coordinate activity in their region. In that case, the SSC and ESAIC will look upon this proposal favourably, which could result in a distinct analysis and manuscript.

### Early termination of the project

As an observational study, premature termination resulting from ethical or safety concerns is exceedingly unlikely.

### Amendments and changes

Only the SSC or persons delegated by the SSC are entitled to amend the protocol. National coordinators (NCs) and local principal investigators (PIs) will receive timely notification of changes and will be required to submit amendments locally. Written documentation of the amendments’ approval will be provided to the sponsor, and substantial amendments to the protocol will be only implemented after necessary local approvals. In consideration of the observational nature of the study, the necessity of protocol deviations to protect the rights, safety, and well-being of human subjects without prior approval of the sponsor and the IRB appears remote. Such deviations must be documented and reported to the sponsor and the IRB as soon as possible.

### Publication of results

The results of SQUEEZE and its sub-studies will be published in peer-reviewed international medical journals and presented at Euroanaesthesia and national meetings. For reporting the results, the Strengthening the Reporting of Observational Studies in Epidemiology (STROBE) statement will be used (Vandenbroucke et al. 2007).

As recommended by the International Committee of Medical Journal Editors, authorship will be considered based on contributions to the recruitment of patients, data acquisition and cleaning, analysis and interpretation of the data, manuscript writing and submission of national/local grants, AND final approval of the version to be published AND agreement to be accountable for all aspects of the work in ensuring that questions related to the accuracy or integrity of any part of the work are appropriately investigated and resolved.

Members of the SSC and other particularly committed investigators (see below) that fulfil those criteria will be part of the writing group. The members of the writing group and the “SQUEEZE Investigators” will be authors of the publications derived from SQUEEZE. When submitting a manuscript, the corresponding author will specify the group name as “SQUEEZE Investigators”. According to the recommendations issued by the International Committee of Medical Journal Editors, the article’s by-line identifies who is directly responsible for the manuscript, and MEDLINE lists as authors and collaborators, whichever names appear on the by-line. To ensure that MEDLINE will list the names of individual group members who are collaborators, there will be a note associated with the by-line stating that the individual names are elsewhere in the paper, and those names are collaborators. The local PI will be asked to submit names of staff actively involved from their institution at the end of study reporting form.

Presentation at international meetings will be restricted to the members of the SSC or their delegates. National coordinators will qualify for presentation at national meetings after approval by the SC and the sponsor. ESAIC Clinical Trial Network will be acknowledged in all publications and presentations.

### Data sharing and secondary analyses

After the pooled results are published, centres will be allowed to use their own data for local presentation and publication. Duplicate data publication is not permitted.

The pseudonymised pooled dataset may be available for secondary analyses upon specific request in the form of a detailed study proposal (including authorship rules) to the SSC. Only collaborators may have access to the study data. The final approval of these potential secondary analyses rests with the SC. Prior to journal submission, any paper originating from the pooled data will be reviewed by the SSC, which is also entitled to require revisions. Authorship of any publication derived from the pooled data set will include the group name “SQUEEZE Investigators” with a by-line clearly stating that the individual names are elsewhere in the paper. For transparency, the original paper has to be referenced in all articles of secondary analyses. Requests for data sharing for individual-level meta-analyses are to be addressed to the sponsor and SC.

## Supplementary Information

**Additional file 1:** **Appendix 3.** LOGISTICS of delivery.

**Additional file 2.** Case Report Form.

**Additional file 3.** Statistical Analysis Plan v4 - FINAL [2021-10-21].

## Data Availability

The pseudonymized pooled dataset may be available for secondary analyses upon specific request in the form of a detailed study proposal (including authorship rules) to the steering committee.

## Abbreviations

AUC: Area under the curve
CI: Confidence interval(s)
CRF: Case report form
eCRF: Electronic case report form
CTN: Clinical Trial Network
EPCO: European Perioperative Clinical Outcome definitions
ESAIC: European Society of Anaesthesiology and Intensive Care
EuSOS: European Surgical Outcome Study
ICH-GCP: International Council for Harmonisation — Good Clinical Practice
ICF: Informed consent form
ICU: Intensive care unit
IRB: Internal review board
NC: National coordinator
OR: Odds ratio
PI: Principal investigator
ROC: Receiver operating characteristics (curve)
RR: Relative risk
SAE: Serious adverse event
SOP: Standard operational procedure
SSC: Study steering committee
MAP: Mean arterial pressure
