# Supplementary material for: The incidence of postoperative vasopressor usage: protocol for a prospective international observational cohort study (SQUEEZE)
Source: Perioper Med (Lond). 2023 Mar 24;12:8. doi: 10.1186/s13741-023-00296-1 (PMC10037774; doi:10.1186/s13741-023-00296-1)
Supplement: Supplementary file 1 — Additional file 1: Appendix 3. LOGISTICS of delivery. [file 13741_2023_296_MOESM1_ESM.docx]

Appendix 3

# LOGISTICS of delivery

## Study centres and role of national coordinators (NC) and local principal investigators (PI)

We aim to recruit as many centres from high- and middle-income countries as possible. We aim to have at least 20 countries. Within each country, we will aim to recruit as many centres as possible. The number of centres will inevitably vary by country. The NC will scrutinise potential participating hospitals to ensure that they will be able to collect the necessary data - guidance will be provided. Each centre should recruit consecutive data from all patients during a seven-day period, followed by 30 subsequent patients that receive postoperative vasopressors. See chapter 5.1. and 5.2 for inclusion/exclusion criteria. Recruiting from low-income countries has previously been found to be challenging due to resource constraints but they will not be excluded from participating.

We aim to meet or exceed this target through the activities of national lead investigators and the support of key organisations such as the European Society of Anaesthesiology and Intensive Care and other supporting societies (ESICM).

*Centres*

Study centre registration occurs online via the dedicated “Call for Centres form” on the ESAIC website. Within the period of recruitment planned for SQUEEZE, the start of recruitment for individual centres (12 months) is at the discretion of the local PI. Recruitment will continue until each centre has recruited all eligible patients for one week plus an additional 30 patients that receive vasopressors or 12 months have passed since starting to recruit cohort B.

*National coordinators*

National coordinating investigators are appointed by ESAIC and the SSC to lead the project within individual countries and their responsibility includes:

• Identify participating centres in their country and recruit local PIs in participating hospitals;

• Assist in the translation of study documents;

• Ensure that all necessary national or regional regulatory approvals are in place prior to start of patient inclusion;

• Assist and train the Local PI and monitor the conduct of the study according to GCP;

• Ensure good communication with ESAIC headquarters and the participating sites in his/her countries during all study steps including data cleaning.

*Principal investigators*

Local PI are specialists working in perioperative medicine in each participating institution who will have the following responsibilities:

- Provide leadership for the study in their institution;
- Ensure all relevant regulatory/ethical approvals are in place for their institution;
- Ensure adequate training of all relevant staff prior to data collection;
- Supervise enrolment, daily data collection, and assist with problem solving;
- Adjudicate events
- Ensure timely completion of eCRF, follow-up assessments, and data cleaning queries. The Local PI is the main responsible for ensuring integrity of data collection. By signing the data on eCRF Local PI confirms the data integrity´;
- Communicate with ESAIC headquarter and the relevant National Coordinating Investigator during all study steps including data cleaning.
