# Supplementary material for: The incidence of postoperative vasopressor usage: protocol for a prospective international observational cohort study (SQUEEZE)
Source: Perioper Med (Lond). 2023 Mar 24;12:8. doi: 10.1186/s13741-023-00296-1 (PMC10037774; doi:10.1186/s13741-023-00296-1)
Supplement: Supplementary file 2 — Additional file 2. Case Report Form. [file 13741_2023_296_MOESM2_ESM.pdf]

Patient ID: |__|__|__|-|__|__|__|-|__|__|__| Sex: Male / Female

CRF 1

0. Informed consent

Is consent applicable in your centre ?<br>*Mandatory unless the centre has an explicit and written exemption from IRB*	o No<br>o Yes	If yes date of consent:	DD-MMM-YY

1. Patient Information

1.1. Year of Birth *		1.2. Weight *		1.3. Height *	

1.4. Clinical Frailty Scale *	1 Very fit	◯	6 Moderately Frail	◯
	2 Well	◯	7 Severely Frail	◯
	3 Managing well	◯	8 Very severely Frail	◯
	4 Vulnerable	◯	9 Terminally	◯
	5 Mildly Frail	◯	10 Don't know	◯

Previous medical history *

1.5. Coronary Artery Disease	o No	o Yes	
1.6. Cerebrovascular Disease	o No	o Yes	
1.7. Peripheral vascular Disease	o No	o Yes	
1.8. Atrial fibrillation	o No	o Yes	
1.9. Heart failure	o No	o Yes	
1.10. Hypertension	o No	o Yes	
1.11. Diabetes	o No	o Insulin dependent	o Non-insulin dependent
1.12. Chronic liver disease	o No	o Yes	
1.13. Chronic respiratory disease	o No	o COPD	o Other
1.14. Long-term steroid use	o No	o Yes	

1.15. Regular medications (tick all that apply, leave blank if not a regular medication)

ACE inhibitor	If ☐ yes →	o took day of surgery	o omitted day of surgery	o unknown
Alpha blocker	If ☐ yes →	o took day of surgery	o omitted day of surgery	o unknown
Angiotensin receptor blocker	If ☐ yes →	o took day of surgery	o omitted day of surgery	o unknown
Beta blocker	If ☐ yes →	o took day of surgery	o omitted day of surgery	o unknown
Calcium channel blocker	If ☐ yes →	o took day of surgery	o omitted day of surgery	o unknown
Diuretic	If ☐ yes →	o took day of surgery	o omitted day of surgery	o unknown
Regular NSAIDs	If ☐ yes →	o took day of surgery	o omitted day of surgery	o unknown

Haemodynamics. Leave blank if not available

Measurements in the past 6 months or at least 12h prior to the operating room, at rest.

1.16. Systolic:	1.17 Diastolic:	1.18 Heart rate:

Reading immediately prior to induction of anaesthesia:

1.19. Systolic:	1.20 Diastolic:	1.21 Heart rate:

Laboratory. Leave blank if not available, indicate which unit

1.22. Creatinine:		mg/dl or µmol/L
1.23. Albumin		g/dL, g/L or µmol/L
1.24. Haemoglobin		g/dL, g/L or mmol/L

2. Surgery

2.1 Reason for surgery *	Infection	◯
	Cancer	◯

Patient ID: |__|__|__|-|__|__|__|-|__|__|__| Sex: Male / Female

	Fracture	◯
	Bleeding	◯
	Other	◯

2.2 Surgical procedure * (select single most appropriate)	Breast	◯	Orthopaedic	◯
	Gynaecological	◯	Plastics / Cutaneous	◯
	Head and neck	◯	Upper gastro-intestinal	◯
	Hepato-biliary	◯	Neurological/spinal	◯
	Kidney / urological	◯	Vascular	◯
	Lower gastro-intestinal	◯	Other	◯
2.3 Severity *	Minor	◯		
	Intermediate	◯		
	Major	◯		

2.4. ASA-PS: *	ASA 1: Healthy person	◯
	ASA 2: Mild systemic disease.	◯
	ASA 3: Severe systemic disease	◯
	ASA 4 Severe systemic disease that is a constant threat to life.	◯
	ASA 5 A moribund person who is not expected to survive without the operation.	◯
2.5. Urgency *	Urgent (includes emergency, expedited, urgent and immediate)	◯
	Not urgent (includes planned/elective)	◯

**3. Operative**				
3.1. Date of anaesthesia induction *	**DD-MMM-YY**			
3.2. Time of anaesthesia induction *	**HH:MM**			
3.3. Date of end of surgery *	**DD-MMM-YY**			
3.4. Time of end of surgery *	**HH:MM**			
3.5. Estimated blood loss (mL) *	<250 o	251-1000 o	1001-3000 o	>3000 o
3.6 /3.7 Lowest intraoperative blood pressure (paired) *	Systolic:		Diastolic:	

3.8. Anaesthesia: * (Tick all that apply)	Volatile	☐
	TIVA	☐
	Sedation without securing airway	☐
	Regional	☐
	Spinal	☐
	Epidural	☐
3.9. Airway *	Endotracheal tube	◯
	Supraglottic airway	◯
	O2 facemask or nasal cannula	◯
3.10. Arterial line *	o No	o Yes
3.11. Central venous line *	o No	o Yes
3.12. Which Intra-operative vasoactive drugs [Tick all that apply]	Atropine	☐
	Akrinor ® (Cafedrin/Theodrenalin)	☐
	Dobutamine	☐
	Dopamine	☐
	Ephedrine	☐
	Epinephrine (Adrenaline)	☐
	Glycopyrronnium	☐
	Metaraminol	☐

	Milrinone	☐
	Nitrates	☐
	Norepinephrine (Noradrenaline)	☐
	Phenylephrine	☐
	Vasopressin or Terlipressin	☐

3.13. Was the patient receiving a vasopressor infusion prior to anaesthesia? *	o No	o Yes

3.14. Fluids and blood products received during surgery:	Crystalloid	(mL)
	Colloid (starch-gelofusine-albumin)	(mL)
	Packed red blood cells	(mL)
	Fresh frozen plasma	(mL)
	Platelets	(mL)
	Whole blood or autotransfusion	(mL)

**4. Post-operative**<br>Following the end of surgery (within 24h):		
4.1. Did the patient receive enteral vasopressors? (i.e. MIDODRINE) *	o No	o Yes
4.2. Did the patient receive **boluses** of vasopressors? *	o No	o Yes
4.3. Did the patient receive an **infusion** of vasopressors? *	o No (Stop here)	o Yes (Continue with 4.3.1 and 4.3.2)
4.3.1. **If yes**, did the infusion continue or start after 1 hour from the end of surgery?	o No	o **Yes**
4.3.2. **If Yes**, Did this infusion start within 24 hours from the end of surgery?	o No	o **Yes**
**If "yes" on 4.3.1 and .4.3.2, complete CRF2**		

**5. Outcomes**			
5.1. Ventilation: *	o No	o Invasive mechanical ventilation (IMV)	o Non Invasive Ventilation (NIV)
5.2. Acute Myocardial Infarction *	o No		o Yes
5.3. New onset atrial fibrillation *	o No		o Yes
5.4. New onset other dysrhythmia *	o No		o Yes
5.5. Renal: Highest creatinine (within the first week) postoperatively			mg/dl or µmol/L indicate which unit
5.6. Renal replacement therapy *	o No		o Yes
5.7. Parenteral nutrition *	o No		o Yes
5.8. Antibiotics for a newly diagnosed infection *	o No	o Yes (complete ⇩)	o Unknown
	o Skin or soft tissue<br>o Respiratory<br>o Urinary		o Abdominal<br>o Lines<br>o Other
5.9. Accordion classification of surgical complication *	o None		
	o Mild complication		
	o Moderate complication		
	o Severe complication		

	○ Death	
5.10. Date of hospital discharge **or** date of intrahospital death: *		DD-MMM-YY
5.11. Stayed an inpatient for more than 30 days? *	○ No	○ Yes

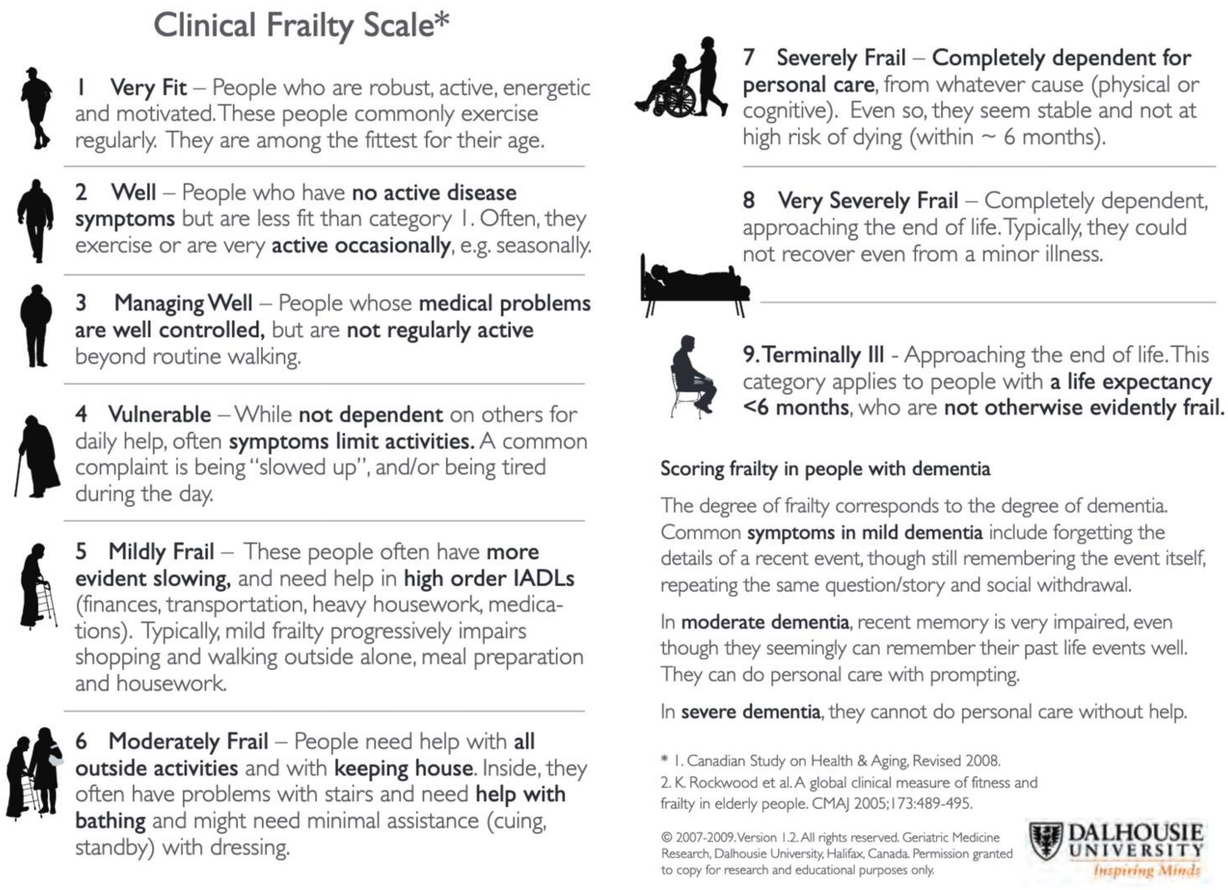

Clinical Frailty Scale*

1 **Very Fit** – People who are robust, active, energetic and motivated. These people commonly exercise regularly. They are among the fittest for their age.

2 **Well** – People who have **no active disease symptoms** but are less fit than category 1. Often, they exercise or are very **active occasionally**, e.g. seasonally.

3 **Managing Well** – People whose **medical problems are well controlled,** but are **not regularly active** beyond routine walking.

4 **Vulnerable** – While **not dependent** on others for daily help, often **symptoms limit activities.** A common complaint is being "slowed up", and/or being tired during the day.

5 **Mildly Frail** – These people often have **more evident slowing,** and need help in **high order IADLs** (finances, transportation, heavy housework, medications). Typically, mild frailty progressively impairs shopping and walking outside alone, meal preparation and housework.

6 **Moderately Frail** – People need help with **all outside activities** and with **keeping house**. Inside, they often have problems with stairs and need **help with bathing** and might need minimal assistance (cuing, standby) with dressing.

7 **Severely Frail** – **Completely dependent for personal care**, from whatever cause (physical or cognitive). Even so, they seem stable and not at high risk of dying (within ~ 6 months).

8 **Very Severely Frail** – Completely dependent, approaching the end of life. Typically, they could not recover even from a minor illness.

9. **Terminally Ill** - Approaching the end of life. This category applies to people with **a life expectancy <6 months**, who are **not otherwise evidently frail.**

Scoring frailty in people with dementia

The degree of frailty corresponds to the degree of dementia. Common **symptoms in mild dementia** include forgetting the details of a recent event, though still remembering the event itself, repeating the same question/story and social withdrawal.

In **moderate dementia**, recent memory is very impaired, even though they seemingly can remember their past life events well. They can do personal care with prompting.

In **severe dementia**, they cannot do personal care without help.

* 1. Canadian Study on Health & Aging, Revised 2008.
2. K. Rockwood et al. A global clinical measure of fitness and frailty in elderly people. CMAJ 2005;173:489-495.

© 2007-2009. Version 1.2. All rights reserved. Geriatric Medicine Research, Dalhousie University, Halifax, Canada. Permission granted to copy for research and educational purposes only.

DALHOUSIE UNIVERSITY *Inspiring Minds*

2.3. Severity of surgery:

<u>Minor</u>: Procedure < 30 minutes. Examples: arthroscopy without intervention, removal of cutaneous tumour, proctology procedures, biopsy or excision biopsy of small lesions, etc

<u>Intermediate</u>: Procedure performed in a dedicated operating room that may pose the risk of significant complications or tissue injury. Examples: laparoscopic cholecystectomy, arthroscopy with intervention, bilateral varicose vein removal, tonsillectomy, inguinal hernia repair, breast lump resection, haemorrhoidectomy, appendicectomy, partial thyroidectomy, cataract surgery, uvuloplasty, minimally invasive repair of vaginal prolapse, vaginal hysterectomy, fixation of mandibular fracture, etc

<u>Major</u>: Performed in a dedicated operating room and is expected to last more than 90 minutes. Examples: major gut resection, major joint replacement, mastectomy, extensive head and neck tumour resection, abdominal aortic aneurysm repair, major vascular bypass procedure, procedures involving free flap to repair tissue defect, amputation, total thyroidectomy, cystectomy, trans-urethral resection of prostate, resection of liver tumour, carotid endarterectomy, nephrectomy, total abdominal hysterectomy, spinal discectomy, etc

CRF 2

**Postoperative vasopressor infusion**		
6.1. Did this patient have an **infusion** of vasopressors that was either started or continued at least 1 hour after surgery: *	o No<br>(If 'No' then please do not complete any further)	o Yes

At one hour after the completion of surgery, is the patient:		
6.2. Receiving continuous infusion of neuraxial anaesthesia/analgesia i.e. epidural infusion *	o No	o Yes
6.3. Still receiving a sedative infusion *	o No	o Yes
6.4. Still has an airway in place (endotracheal tube, tracheostomy or supraglottic airway) *	o No	o Yes

6.5. How was it assessed that this patient should receive a vasopressor infusion? *

- o Already receiving a vasopressor infusion and attempts to lower the infusion rate produced unacceptable hypotension, OR
- o It was decided that the patient would no longer benefit from further attempts to increase the cardiac output through administration of IV fluids and the blood pressure was unacceptably low. This was on the basis of:
  - o A. Clinical assessment alone (vital signs-examination-lab results)
  - o B. Clinical assessment AND a measurement of preload responsiveness using cardiac output monitoring (or some direct surrogate of)
  - o C. Clinical assessment AND a measurement of preload responsiveness using echocardiography
  - o D. Clinical assessment AND a previously established maximum for IV fluid administration has been met i.e. 2L or 20ml/kg etc...
  - o E. other:

7.1. SOFA score within 24 hours after surgery * [0-24]		(Use FAQ as required)<br>To calculate SOFA score:<br>https://clincalc.com/IcuMortality/SOFA.aspx

7.2 - 7.8 MAP target (complete only if MAP is specified)

	Day 0	Day 1	Day 2	Day 3	Day 4	Day 5	Day 6
MAP							

7.10 – 7.16 **HIGHEST** blood pressure for each day (paired)
On Postoperative unit/ICU only. Leave blank if not available.

	Day 0	Day 1	Day 2	Day 3	Day 4	Day 5	Day 6
Systolic							
Diastolic							

7.18 – 7.24 **LOWEST** blood pressure during the day (paired)<br>On Postoperative unit/ICU only. Leave blank if not available.							
	Day 0	Day 1	Day 2	Day 3	Day 4	Day 5	Day 6
Systolic							
Diastolic							

7.26 – 7.32 **Vasoactive drug infusion, tick if applicable**							
	Day 0	Day 1	Day 2	Day 3	Day 4	Day 5	Day 6
Noradrenaline	☐	☐	☐	☐	☐	☐	☐
Angiotensin II	☐	☐	☐	☐	☐	☐	☐
Dobutamine	☐	☐	☐	☐	☐	☐	☐
Dopamine	☐	☐	☐	☐	☐	☐	☐
Epinephrine (Adrenaline)	☐	☐	☐	☐	☐	☐	☐
Metaraminol	☐	☐	☐	☐	☐	☐	☐
Milrinone	☐	☐	☐	☐	☐	☐	☐
Phenylephrine	☐	☐	☐	☐	☐	☐	☐
Terlipressin	☐	☐	☐	☐	☐	☐	☐
Vasopressin	☐	☐	☐	☐	☐	☐	☐

Total number of days: *	
7.33. receipt of ventilation (invasive or NIV)	
7.34. receipt of vasopressor infusion	
7.35. receipt of parenteral nutrition	
7.36. receipt of renal replacement therapy	
7.37. duration of stay in ICU/postoperative unit	

COVID			
7.38. Did the patient have any testing for SARS-CoV2? *	o No	o Yes<br>**If yes, answer below**	o Unknown
7.38.1. **If Yes -** did the patient test positive in the perioperative period?	o No	o Yes	
