# Supplementary material for: The incidence of postoperative vasopressor usage: protocol for a prospective international observational cohort study (SQUEEZE)
Source: Perioper Med (Lond). 2023 Mar 24;12:8. doi: 10.1186/s13741-023-00296-1 (PMC10037774; doi:10.1186/s13741-023-00296-1)
Supplement: Supplementary file 3 — Additional file 3. Statistical Analysis Plan v4 - FINAL [2021-10-21]. [file 13741_2023_296_MOESM3_ESM.pdf]

Postoperative vasopressor usage: a prospective international observational study
Statistical Analysis Plan

Final agreed version - v4, 21 October 2021

Peter Martin, University College London

Aims

Squeeze is an international multi-centre prospective observational study on the use of postoperative vasopressors after surgery in Europe.

The aims of the study are:

1. Prevalence of use of Postoperative Vasopressor Infusions (PVI):
    a. To estimate the proportion of patients who receive postoperative vasopressor infusions in Europe
    b. To estimate the between-hospital variation of patients receiving PVIs
2. Predictors of PVI use: To investigate patient characteristics that predict the use of PVI
3. Outcomes after PVI: to investigate the association between use of PVI and patient outcomes: organ dysfunction, length of stay, critical care stay, and in-hospital mortality
4. To document the distributions of PVI type and dose in patients receiving PVI
5. To document the distribution of duration of PVI use in patients receiving PVI
6. To investigate predictors of prolonged PVI use
7. To explore the associations between prolonged PVI use and outcomes (organ dysfunction, length of stay, critical care stay, and in-hospital mortality)

Sample

Squeeze will collect data on two cohorts of patients, as outlined in the study protocol.

Cohort A will consist of adult patients undergoing non-day case surgery (but not cardiac, obstetric, or transplant surgery). Hospitals will be instructed to enter all eligible patients over a pre-specified one-week period.

Cohort B will consist of adult patients undergoing non-day case surgery (but not cardiac, obstetric, or transplant surgery) who receive PVIs. Hospitals are instructed to enter all eligible patients over a period of 12 months excluding the week of during which Cohort A is collected, but to halt data collection when a sample of 30 patients for cohort B has been reached.

Data Collection

Two case report forms (CRFs) are used.

CRF1 collects data on pre-operative patient characteristics, type of surgery, intraoperative variables, post-operative patient condition and post-operative treatment, including whether PVI was used in the period following one hour after surgery. CRF1 should be completed for all patients in Cohorts A and B.

CRF2 collects data on reason for PVI use, as well as type, dose, and duration of PVI use for up to six days following the surgery. CRF2 should be completed for all patients in Cohort B, and those patients in Cohort A who receive PVI.

(UK hospitals collect additional data via a third CRF. These additional data will be analysed in a separate project.)

Statistical analysis

Preliminary considerations

This is an observational study based on a self-selected set of hospitals. Although our sampling procedures give us a good chance of achieving a representative sample of patients within each participating site, we do not claim to be able to achieve a random sample of hospitals from participating countries, or a representative sample of patients for any country as a whole. Thus, thorough description and graphical representation of the data will be important methods of analysis, and often take precedence over inferential procedures. Some statistical models will be employed to aid description and estimation of essential parameters, as outlined below. We will summarize patient characteristics using means, standard deviations, medians, interquartile ranges, and percentages as appropriate.

In general, estimation of the association between PVI use and outcomes from these data won't enable us to estimate an *effect* of PVI on these outcomes, since there is likely to be confounding by indication. However, the sizes of the associations found may be informative for future studies that wish to rigorously investigate the effect of PVI on outcomes, including randomised controlled trials.

Aim 1: Prevalence of PVI use and between-hospital variation

This analysis will use patients from Cohort A only. The estimand is the proportion of patients from the eligible population who receive PVI. Since hospitals enter the study by self-selection, we won't be able to ascertain whether we can obtain an unbiased estimate of this quantity. We will report the crude proportion of patients receiving PVIs in our data set as an approximate estimate. We will also document the distribution of the proportion of patients receiving PVI by hospital.

Initially we will estimate the rate of PVI use across all countries. The proportion of PVI use will also be reported separately for all countries. Exploratory analyses will determine how we further summarise and present the data. This involves establishing whether the difference in rate of PVI use between low and middle income countries (LMICs) and high-income countries is large relative to the variation between countries overall. If this difference is small, we will use data from all countries together for subsequent analyses. If the difference is large, we will select high-income countries for subsequent analyses. Under these circumstances, LMICs would be reported separately.

Mixed effects logistic regression will be used to document between-hospital variation, employing a shrinkage estimator (best linear unbiased prediction) on the hospital-specific log odds of PVI use to control for regression to the mean. A caterpillar plot will illustrate the distribution. The assumption of normality of hospital-level random intercepts will be assessed.

If substantive between-hospital and/or between-country variation is found, we will explore the association between this variation and reasons given for PVI use in CRF items 5.5 and 5.6. This will be a descriptive analysis only.

Aim 2: Predictors of PVI use

This analysis will use all patients from Cohorts A and B. We will estimate two models, which differ in the sets of potential predictors considered:

Model 1: Use pre-operative variables as predictors only.
Model 2: Use both pre-operative and intra-operative variables as predictors.

In Model 2 only, we will explore whether results differ if we analyse data separately for the following subgroups:

a) Patients in receipt of epidural anaesthesia
b) Patients in receipt of spinal anaesthesia
c) All other patients

The variables to be considered as candidate predictors of PVI use in each model are specified in Appendix A. All predictor variables will be described using standard descriptive statistics.

The causal relationships between these predictors and PVI use are a priori uncertain. Some potential predictor variables may be on the causal path between other predictors and the probability of PVI use, but we do not know this in advance. The aim of this analysis is to document associations to inform future studies.

Predictors of PVI use will be assessed via a mixed effects logistic regression, with a random intercept term to account for baseline variation in PVI use between hospitals. A random intercept term for countries will also be explored. The adaptive lasso estimator will be employed to shrink slope estimates relating to predictor variables. This reduces the risk of overfitting in the presence of many predictor variables. The adaptive lasso also results in

predictor selection, and thus provides a model-based method to simplify the presentation of results and to concentrate on the most important predictors.

For continuous predictors, fractional polynomials will be used prior to estimating a full model in order to determine the best way to model the relationship between each continuous predictor and the probability of PVI use.

Aim 3: Patient outcomes of PVI use

This analysis will use all patients from Cohorts A and B. The following post-operative outcomes will be considered. Numbers in brackets refer to the variable number in the case report form (CRF1).

- Indicators of organ dysfunction
  - Ventilation (5.1): invasive mechanical, non-invasive mechanical, none (3 categories)
  - Acute myocardial infarction (5.2): yes, no
  - New onset of atrial fibrillation (5.3): yes, no
  - New onset of other dysrhythmia (5.4): yes, no
  - Presence of acute kidney injury (AKI), indicated by change in creatinine from documented preoperative baseline (CRF item 5.5 in relation to 1.22):
    - increase<50 %: no AKI
    - 50%<= increase< 100 %: stage 1,
    - 100%<= increase< 200 %: stage 2,
    - increase >= 200%: stage 3
  - Renal replacement therapy (5.6): yes, no
  - Parenteral nutrition (5.7): yes, no
  - Antibiotics for newly diagnosed infection (5.8): yes, no
  - Severity of complication (5.9): none, mild, moderate, severe, death (5 categories)
- 30-day in-hospital mortality (5.10 and 5.10.2)
- Length of hospital stay from time of operation (5.10, 5.10.1, and 5.11) for those who were discharged alive

All analyses will establish the unadjusted association between PVI use and each outcome. Additionally, an adjusted model will be estimated, controlling for the pre-operative predictors of PVI use established in the analyses under Aim 2, Model 1. Different types of mixed effects regression models will be used depending on the outcome concerned, as specified below.

30-day in-hospital mortality and dichotomous organ failure indicators: A mixed effects logistic regression will be estimated to assess the evidence for an association between PVI use and the probability of adverse outcome.

Ordered categorical organ failure indicators: A mixed effects generalized ordered logit model will be estimated to assess the evidence for an association between PVI use and severity of complications, presence of AKI, and ventilation, respectively.

Length of stay: A mixed effects quantile regression predicting the quartiles of the length of stay distribution will be estimated to assess the evidence for an association between PVI use and length of stay.

Aim 4: Type and dose of PVI

This analysis will include all patients receiving PVI (some from Cohort A and all from Cohort B). We will describe the proportion of PVI patients who receive each of eight types of vasoactive drugs (norepinephrine, phenylephrine, etc.). For each drug, we will describe the distribution of dosages, separately by day since surgery, using standard descriptive statistics.

Aim 5: Duration of PVI use

This analysis will include all patients receiving PVI (some from Cohort A and all from Cohort B). We will document the distribution of duration of PVI use, measured in days from day of surgery up to day 6 after surgery. We will also document the between-hospital variation in duration of PVI use. A suitable model for doing so will be determined after exploratory analysis, depending on the distribution of the duration variable. For example, duration could be modelled as a count variable, as a dichotomised variable, or via quantile regression.

Aim 6: Predictors of prolonged PVI use

This analysis will include all patients receiving PVI (some from Cohort A and all from Cohort B). We will use a generalised linear mixed model or a mixed effects quantile regression to examine the association between pre- and intra-operative patient characteristics and prolonged PVI use. A suitable model for the duration variable, including what constitutes 'prolonged PVI use', will be determined by exploratory analysis, depending on the results under Aim 5 (see above). The list of potential candidate predictor variables for this model can be found in Appendix A. We will use adaptive lasso shrinkage to reduce the risk of overfitting, and for predictor selection.

Aim 7: Association between prolonged use of PVI and outcomes

This analysis will include all patients receiving PVI (some from Cohort A and all from Cohort B). The following post-operative outcomes will be considered:

- Indicators of organ dysfunction
    - Ventilation (3 categories: invasive mechanical, non-invasive mechanical, none)
    - Acute myocardial infarction (yes, no)
    - New onset of atrial fibrillation (yes, no)
    - New onset of other dysrhythmia (yes, no)
    - Renal replacement therapy (yes, no)
    - Presence of acute kidney injury (AKI; see under Aim 3 for specifications of this outcome)
    - Parenteral nutrition (yes, no)
    - Antibiotics for newly diagnosed infection (yes, no)
    - Severity of complication (5 categories: none, mild, moderate, severe, death)
- 30-day in-hospital mortality

We will document the unadjusted associations between PVI use and length of stay, surgical complication score, and 30-day in-hospital mortality. To do this, we will conduct a descriptive analysis of variations in three outcomes by the extent of intra- and post-operative vasopressor use, according to the following schema:

	Vasopressor usage					
	None	Intraop. boluses or infusion only	Postop. boluses only	Both intra- and postop boluses	PVI	Prolonged PVI
Length of stay (quartiles)						
Surgical complication score (quartiles)						
30-day in-hospital mortality (proportion)						

In doing so, we will carefully document the time-to-death of all patients who died in hospital, and thus describe whether the relationships observed in the table above may be due to biases associated with early deaths, for example because patients who died soon after the operation by definition may not have experienced prolonged PVI use.

Missing values

Most potential predictors of PVI use are assessed via required fields in the CRF, so item-level missing values cannot arise there by definition. Non-required fields are variables relating to haemodynamics and laboratory blood measurements, as well as fluids and blood products received during surgery. Missing values will be documented and potential bias arising from a complete cases analysis will be assessed from the data. Multiple imputation of missing values will be considered if the missing at random assumption is judged to be likely to be met.

Appendix A: Candidate predictor variables of PVI use

This appendix lists variables considered as candidate predictors of PVI use in the analyses specified under Aim 2. We separate pre-operative predictors and intraoperative predictors. Numbers in brackets refer to the variable number in the CRF.

Pre-operative predictors (Model 1 in Aim 2)

- Age (1.1)
- Clinical Frailty Scale (1.2)
- Previous medical history (1.5, 1.6, 1.7, 1.8, 1.9, 1.10, 1.11, 1.12, 1.13, 1.14);
  - We will consider grouping coronary, cerebrovascular and peripheral vascular disease together as "atherosclerotic disease"
- Regular medications (1.15)
- Mean Arterial Pressure (MAP), calculated as MAP = [SBP + 2DBP]/3
  - At least 12 hrs before operation (1.16, 1.17)
  - Pre-anaesthesia (1.19, 1.20)
- Reason for surgery (2.1, 5 options)
- Surgical procedure type (2.2, 12 options)
- Severity (2.3, 3 options)
- ASA-PS (2.4, 5 options)
- Urgency (2.5, 2 options)
- Duration of anaesthesia/operation (3.3/3.4 minus 3.1/3.2)
- Estimated blood loss (3.5)
- Lowest intraoperative BP (3.6/3.7)
- Type of anaesthesia (3.8), comparisons:
  - 'volatile + TIVA' (without others) vs. regional/epidural/spinal (without others) vs. sedation (without others)
  - volatile (without others) vs. TIVA (without others)
- Airway subglottic/ETT/neither (3.9)

Intraoperative predictors

- Intraoperative vasopressor (3.12)
- Pre-op vasopressor (3.13)
- Volume of fluids given (3.14)
  - Cumulative of all types of fluid
  - Crystalloid vs. colloid
  - Blood products (PRBC, FFP, PLT) versus others
- Enteral vasopressors yes/no (4.1)
- Boluses of post-op vasopressors yes/no (4.2)
